# Scarless and sequential gene modification in *Pseudomonas *using PCR product flanked by short homology regions

**DOI:** 10.1186/1471-2180-10-209

**Published:** 2010-08-03

**Authors:** Rubing Liang, Jianhua Liu

**Affiliations:** 1 School of Life Science & Technology, Shanghai Jiao Tong University, 800 Dong-Chuan Road, Shanghai 200240, China

## Abstract

**Background:**

The lambda Red recombination system has been used to inactivate chromosomal genes in various bacteria and fungi. The procedure consists of electroporating a polymerase chain reaction (PCR) fragment containing antibiotic cassette flanked by homology regions to the target locus into a strain that can express the lambda Red proteins (Gam, Bet, Exo).

**Results:**

Here a scarless gene modification strategy based on the Red recombination system has been developed to modify *Pseudomonas *genome DNA via sequential deletion of multiple targets. This process was mediated by plasmid pRKaraRed encoding the Red proteins regulated by *P*_*BAD *_promoter, which was functional in *P. aeruginosa *as well as in other bacteria. First the target gene was substituted for the *sacB*-*bla *cassette flanked by short homology regions (50 bp), and then this marker gene cassette could be replaced by the PCR fragment flanking itself, generating target-deleted genome without any remnants and no change happened to the surrounding region. Twenty genes involved in the synthesis and regulation pathways of the phenazine derivate, pyocyanin, were modified, including one single-point mutation and deletion of two large operons. The recombination efficiencies ranged from 88% to 98%. Multiple-gene modification was also achieved, generating a triple-gene deletion strain PCA (PAO1, *ΔphzHΔphzMΔphzS*), which could produce another phenazine derivate, phenazine-1-carboxylic acid (PCA), efficiently and exclusively.

**Conclusions:**

This lambda Red-based technique can be used to generate scarless and sequential gene modification mutants of *P. aeruginosa *efficiently, using one-step PCR product flanked by short homology regions. Single-point mutation, scarless deletion of genes can be achieved easily in less than three days. This method may give a new way to construct genetically modified *P. aeruginosa *strains more efficiently and advance the regulatory network study of this organism.

## Background

Obtainment of the genome sequences of more and more bacteria have provided researchers a wealth of information to restructure custom-designed microbes for therapeutic and industrial applications [1-3]. One of the most common approaches is sequence-specific deletion or insertion of the target genes or DNA fragments, and various methods have been developed based on the RecA-independent homologous recombination, such as RecET and lambda Red recombination system [4-8]. In these recombination events, selection markers, usually antibiotic markers are needed to confirm the modification procedure, which may have influence on further manipulation. To solve this problem, the Flp/FRT and Cre/loxP site-specific recombination systems have been used for the precise excision of selection markers. However, even combined with these systems, one copy of FRT or loxP site still remains on the genome after excision [9,10].

*P. aeruginosa *is a gram-negative opportunistic human pathogen of growing clinical importance. The sequence analysis on the 6.3 Mb genome of *P. aeruginosa *PAO1 revealed 5700 predicted open reading frames (ORF) [11]. Many genetic tools have been developed for its genome-scale and proteome-scale research, such as commercial (Affymetrix, Santa Clara, CA) *P. aeruginosa *GeneChips^® ^for transcriptome analysis and the transposon mutants library for sequence-defined mutants [12-15]. Almost in all of these methods, it is necessary to use the suicide vector and the conjugation transfer to isolate the defined mutant, which is a quite tedious process. In addition, to make unmarked deletion mutants, researchers have developed several methods combining the counter-selectable markers (*sacB*) with the site-specific Flp or Cre recombinase [16,17]. However, these methods can not generate the true "scarless" mutants.

Here a two-step approach was described to perform the scarless and sequential genome modification using one-step PCR product with short (50 bp) homology regions. The homologous recombination process was mediated by an RK2-derived plasmid, pRKaraRed, expressing the genes of lambda-Red system (*gam*, *bet *and *exo*) from the arabinose-inducible *P*_*BAD *_promoter. Single gene modification could be finished in three days and the efficiency is higher than 88%. Twelve scarless deletion mutants of different genes, two deletion mutants of large operons, and one single-point mutation were successfully constructed. Furthermore a strain PCA (PAO1, *ΔphzHΔphzMΔphzS*) with deletions in three genes was also generated, which could produce the phenazine-1-acid exclusively and efficiently. This strategy may simplify the genetic manipulation to *P. aeruginosa *and fasten relevant research.

## Results

### Lambda Red-mediated scarless gene modification in *P. aeruginosa*

**Figure 1:**
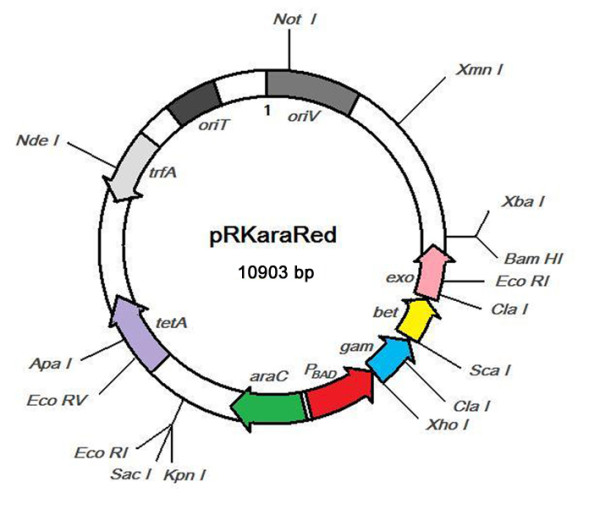
**Map of plasmid pRKaraRed**. Some restriction sites are shown. *tet*A is the tetracycline resistance gene for plasmid selection in *E. coli *and in *P. aeruginosa*. o*riT *is a region for plasmid transfer in *P. aeruginosa*. Expression of lambda Red genes (*gam*, *bet *and *exo*) driven by *P*_*BAD *_promoter are regulated by repressor AraC. The nucleotide sequence of pRKaraRed was deposited in GenBank under the accession number GU186864.

The map of plasmid pRKaraRed was shown in Fig. 1. The backbone was originated from pDN18, in which the *oriV *and *trfA *regions were used to support the plasmid replication and stable maintenance in *P. aeruginosa*, *oriT *region was considered functional for the conjugal transfer among any gram-negative bacterial host virtually and *tetA *was a tetracycline resistance gene [18-20]. The *P*_*BAD *_promoter was used to regulate the expression of lambda Red proteins (Gam, Bet, Exo). The nucleotide sequence of plasmid pRKaraRed was deposited in GenBank under the accession number GU186864.

Initially, *phzS *was selected as target because the phenotype of the mutant could be differentiated from that of the wild type by its inability to produce the pseudomonas blue phenazine pigment, pyocyanin, lack of which resulting a yellowish culture.

**Figure 2:**
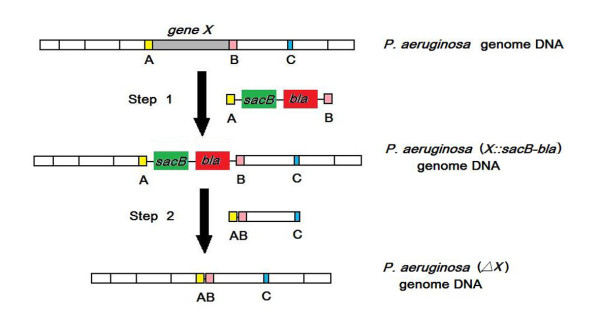
**Schematic description of the scarless gene modification approach**. The first-step of homologous recombination would substitute the genomic target gene *X *for the PCR-amplified *sacB-bla *cassette flanked by the A and B homology regions. The transformants were screened on LB plates containing Carb (500 μg/ml) and Tet (50 μg/ml). The second-step of recombination would replace the *sacB-bla *cassette with PCR-amplified fragments flanked by the AB and C homology regions. As a result, strain with deleted gene *X *and without any remnant on chromosome DNA would be obtained. The transformants of this step were selected on LB plates containing 10% sucrose.

Scarless gene modification could be achieved in two steps (Fig. 2). First the *sacB*-*bla *cassette flanked by short homology regions A and B adjacent to the target was amplified and electro-transformed into the PAO1/pRKaraRed competent cells. Positive colonies (Carb^R^Tet^R^) were then electro-transformed to delete the markers with the *sacB*-*bla *removal cassette, which contained the upstream homology region A and the downstream homology region from B to C (~1000 bp). And the Suc^R^Carb^S ^colonies were regarded as positive recombinants.

The *P*_*BAD *_promoter on plasmid pRKaraRed could be induced by L-arabinose and then the lambda Red proteins could be expressed efficiently, endowing the PAO1/pRKaraRed cells with recombination capability. We first assessed whether 50 bp homology was sufficient to enable efficient homologous recombination between the target and the PCR cassette, which is generally sufficient in *E. coli *[7]. Results showed that the recombination reactions with 1×10^9 ^cells and aliquots of 1 or 2 μg electroporated PCR products could generate 30~80 Carb^R ^transformants, and the colonies number would double approximately when 4 μg DNA was used. Controls (uninduced cells, induced cells without plasmid, and induced cells without DNA fragments) have no transformants. Then the insertion of the *sacB-bla *cassette and the pyocyanin producing ability of all the Carb^R ^colonies were analyzed. And almost all the colonies were positive recombinants (Table 1). The recombination reactions using PCR cassettes flanked by other length of homology regions (60 bp and 100 bp) were also performed, and the recombination efficiency was slightly increased (Table 1). Therefore, 50 bp homology was enough to promote the efficient homologous recombination.

**Table 1:** Efficiencies of pRKaraRed-mediated recombination under different conditions

Conditions	**Positive colonies/Growing colonies (%)**^**a**^		Overall efficiency (%)
		
	**Replacement with marker genes**^**b**^	**Deletion of marker genes**^**c**^	
A. L-arabinose concentration			

0.05%	10/19 (53%)	9/20 (45%)	24%

0.1%	31/43 (72%)	17/20 (85%)	61%

0.2%	67/68 (99%)	20/20 (100%)	99%

0.4%	62/63 (98%)	20/20 (100%)	98%

0.8%	70/73 (96%)	20/20 (100%)	96%

1.0%	59/61 (97%)	19/20 (95%)	92%

B. Length of homology regions			

50 bp	66/67 (99%)	20/20 (100%)	99%

60 bp	72/73 (99%)	20/20 (100%)	99%

100 bp	79/80 (99%)	20/20 (100%)	99%

C. Induction time			

1 hours	33/39 (85%)	17/20 (85%)	72%

3 hours	63/64 (98%)	20/20 (100%)	98%

6 hours	56/57 (98%)	20/20 (100%)	98%

12 hours	48/49 (98%)	19/20 (95%)	93%

*phzS *gene was used as target. Conditions: A, 50 bp homology region, induction of cells with different concentration of L-arabinose during 3 hours; B, different lengths of homology regions, induction of cells with 0.2% L-arabinose during 3 hours; C, 50 bp homology region, induction of cells with 0.2% L-arabinose during different time.

a. Determined by PCR amplification and DNA sequencing

b. Screening of Carb^R^Suc^S ^colonies

c. Screening of Carb^S^Suc^R ^colonies

The influences of the L-arabinose concentration and the induction time on the recombination efficiency were also analyzed. Results indicated that when the concentration of L-arabinose went up, the recombination efficiency also increased gradually which could reach the maximum at the concentration of 0.2% and keep stable. Induction time also had influence on the recombination efficiency and efficient recombination could be achieved after the cells were induced with 0.2% L-arabinose for at least three hours (Table 1).

### Gene modifications in *P. aeruginosa *PAO1

**Figure 3:**
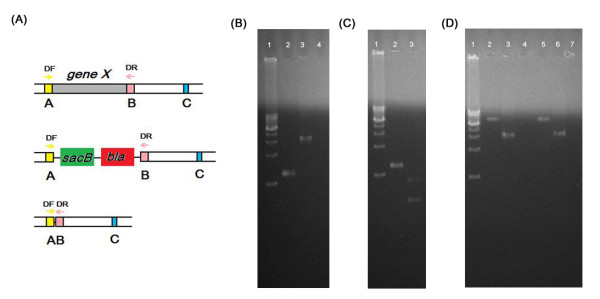
**Plasmid pRKaraRed mediated scarless gene modification to PAO1 genome**. (A). The scheme of the scarless gene modification. Primers DF and DR were used to verify the substitutions of target fragments. (B). PCR results of *phzS *deletion detected using primers phzS-DF and phzS-DR. Lanes: 1, DNA marker (Takara 1 kb marker, from 1.0 kb to 10.0 kb); 2, the PCR product of *phzS *gene; 3 and 4, the PCR fragments corresponding to the recombination step 1 and step 2. (C). PCR results of the single-point mutation. Lanes: 1, DNA marker (as mentioned above); 2, the PCR product of *phzS *gene; 3, the *Bam *HI treated PCR fragment after the recombination of two steps. (D) PCR detection results of two operons deletions. Lanes: 1, DNA marker (as mentioned above); 2, the PCR product of *phzA1G1 *operon; 3 and 4, the PCR fragments corresponding to the recombination step 1 and step 2. The PCR amplifications were performed using primers phzA1G1-DF and phzA1G1-DR. Lanes: 5, the PCR product of *phzA2G2 *operon; 6 and 7, the PCR fragments corresponding to the recombination step 1 and step 2. The PCR amplifications were performed using primers phzA2G2-DF and phzA2G2-DR.

Using this pRKaraRed mediated strategy, several mutants were constructed, including twelve deletion mutants of different genes, two deletion mutants of large operons, and one single-point mutation. And the length of modified regions ranged from 1 bp to 6.3 kb (Table 2, Fig. 3). These twelve genes were involved in the synthesis and regulation of pyocyanin and the two operons were the pyocyanin synthesis operons. The point mutation was made at the site 761 of the *phzS *gene, changing the nucleotide A to T, which could produce a *Bam *HI restriction site. Typically 2 μg DNA was electroporated into the PAO1/pRKaraRed competent cells and about 26~78 colonies (Carb^R^Tet^R^) could be obtained. The recombinant efficiencies were about 94~99%, no significant correlation to the size of target gene (Table 2). After the second-step recombination and the sucrose counter-selection, nearly all of the survival colonies were positive recombinants. Indeed, as summarized in Table 2, the overall efficiency of the scarless deletion process ranged from 88% to 98%.

**Table 2:** Efficiencies of pRKaraRed-mediated scarless modification to different targets

Target	Size (bp)	**Positive colonies/Growing colonies (%)**^**a**^		Overall efficiency (%)
			
		**Replacement using *sacB-bla *cassette**^**b**^	**Deletion of *sacB-bla *cassette**^**c**^	
A. Deletion of genes				

*rsm A*	186	43/44 (98%)	19/20 (95%)	93%

*las I*	606	53/54 (98%)	20/20 (100%)	98%

*gac A*	645	49/50 (98%)	18/20 (90%)	88%

*qsc R*	714	36/37 (97%)	19/20 (95%)	92%

*las R*	720	56/57(98%)	20/20 (100%)	98%

*rhl R*	762	59/61(97%)	20/20 (100%)	97%

*phz M*	1005	65/68 (96%)	19/20 (95%)	91%

*rpo S*	1005	46/47 (98%)	20/20 (100%)	98%

*phz S*	1209	70/72 (97%)	20/20 (100%)	97%

*phz H*	1833	68/69 (99%)	19/20 (95%)	89%

*rpo D*	1854	52/54 (96%)	20/20 (100%)	96%

*pts P*	2280	78/80 (98%)	19/20 (95%)	93%

B. Single-point mutation				

*phz S*	1	24/26 (94%)	19/20 (95%)	89%

(A761T)				

C. Deletion of operons				

*phz A1-G1*	6267	47/50 (94%)	19/20 (95%)	89%

*phz A2-G2*	6273	61/63 (97%)	20/20 (100%)	97%

a. Determined by PCR amplification and DNA sequencing

b. Screening of Carb^R^Suc^S ^colonies

c. Screening of Carb^S^Suc^R ^colonies

### Sequential gene deletion and construction of strain PCA

**Figure 4:**
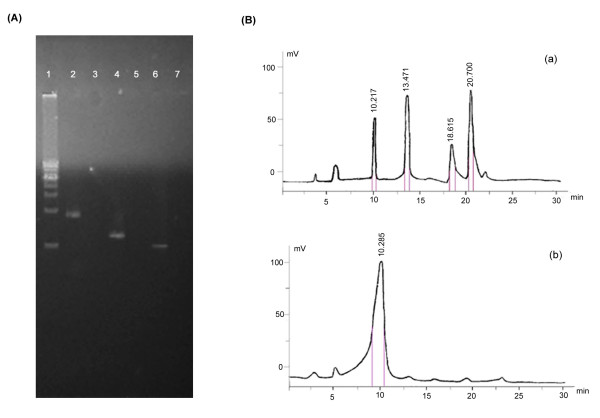
**Sequential deletion of three genes (*phzH*, *phzM *and *phzS*) and the HPLC analysis of phenazine derivatives in *P. aeruginosa *cultured media**. (A). PCR detection results of strain PAO1 and strain PCA. Lane 1 was DNA marker (Takara 1 kb marker, from 1.0 kb to 10.0 kb). Lanes 2, 4, 6 showed the PCR products with the PAO1 genome DNA as template, and lanes 3, 5, 7 showed those using the PCA genome DNA as template. Lanes 2 and 3, 4 and 5, 6 and 7 corresponded to PCR fragments obtained from the pair primers phzH-DF and phzH-DR, phzM-DF and phzM-DR, phzS-DF and phzS-DR, accordingly. (B). The HPLC results of the extracted phenazine derivatives from the cultured media of *P. aeruginosa *PAO1 (a) and *P. aeruginosa *PCA (b). The retention times were shown on the tops. MS was used to identify each fraction collected between the pink lines under the peak. Their chemical identities were PCA (9.17 min-10.43 min), PYO (13.42 min-13.93 min), PCN (18.34 min-18.97 min), and 1-OH-Phz (20.42 min-20.93 min). Four phenazine derivates were detected in the cultured media of strain PAO1, while only PCA was detected in those of PCA strain.

Two-step homogeneous recombination was required for the modification of each gene and the modifications of multiple genes could be easily achieved after several rounds. On this basis, sequential deletion of two, three and four genes were performed successfully. The construction of strain PCA with deletions in three genes, *phzH*, *phzM *and *phzS*, was shown as an example. Proteins PhzS, PhzH and PhzM are involved in the conversion of phenazine-1-carboxylic acid (PCA) into 1-hydroxyphenazine (1-OH-PHZ), phenazine-1-carboxyamide (PCN) and pyocyanin (PYO) [17]. After three rounds of the two-step recombination, these three genes were deleted sequentially and scarlessly (Fig. 4A). As revealed by the HPLC analysis, at least four phenazine derivatives could be detected from the cultured media of PAO1 strain, corresponding to the PCA, 1-OH-PHZ, PCN and PYO, respectively. In the HPLC plot of PCA strain, only one peak representing PCA was detected, and the yield of PCA was higher than that of PAO1 strain (Fig. 4B), indicating that strain PCA could produce PCA efficiently and exclusively.

## Discussion

Lambda Red recombination system first described in *E. coli *has been successfully applied to *Yersinia*, *Salmonella*, *Shigella *and *Serratia *[6,7,21-25]. The procedures involve the homologous recombination between the region of interest and a PCR product containing antibiotic cassette flanked by homology region. Although this efficient method may be applicable to other bacteria, adaptations are frequently required, such as the homology length and recombination steps [22].

In *P. aeruginosa*, construction of markerless deletion mutants is still a time-consuming and labor-intensive process. Two different plasmids were used in the traditional procedure. The first plasmid was transformed for targeting a selected region and the second plasmid was re-transformed for the unmarked deletion of the antibiotic cassette by Flp recombinase [16]. This recombination procedure including multiple steps needs several days to accomplish one gene modification and the recombination efficiency is not very high. Furthermore, the produced "unmarked" deletion is not scarless, as normally one FRT site was left. In 2008, lambda Red system and three-step PCR products were used to replace the target gene with antibiotic cassette in *P. aeruginosa *PA14, which confirmed the possibility of using the lambda Red recombination system in *P. aeruginosa *[26]. However, this method can not produce scarless modification as the antibiotic cassette is still left on the genome DNA, quite difficult to perform multiple genes modification in one cell. And its homology regions were quite long, meaning several rounds of PCR amplification and more manipulation steps were needed.

As previously reported, multi-copy Red plasmid pTP223 failed to promote gene replacement using the PCR-generated substrates with short homology extensions in *E. coli*, since the linear multimers of this plasmid generated through high dosage of lambda Gam protein drove the plasmid replication in rolling circle mode may be toxic to *E. coli *host or compete with the recombination substrates [27-30]. Based on these observations, we constructed plasmid pRKaraRed derived from RK2, low-copy and broad-host-range expression. As expected, plasmid pRKaraRed was able to promote efficient homologous recombination with short homology extension in *E. coli*, in *P. aeruginosa *PAO1, and also in *Pseudomonas sp. *M18 (data not shown). In *E. coli*, PCR cassettes flanked by only 35 bp homology region could induce the homologous recombination and efficient recombination happened when the PCR fragments flanked by 40 bp homology regions were used (data not shown). But in *Pseudomonas *PAO1 and M18, almost no transformant could be obtained using the PCR fragments with 35 bp or 40 bp homology extension, and at least 50 bp homology regions were required for efficient recombination (30~80 transformants). This is consistent with previous results that the minimum length of homology extension required for efficient recombination may be different when the lambda Red system is used in different organisms, which may have relevance to the characteristics of the organisms, such as the difference in GC content and so on [22-25]. Although the efficiency of recombination in *Pseudomonas *was lower than that in *E. coli*, plasmid pRKaraRed was still suitable for the gene modification in *Pseudomonas*. Differences in the expression of Red proteins, DNA uptake, sequence contexts and the species-specific restriction may result in the variations of recombination efficiency [27].

The scarless modification strategy based on plasmid pRKaraRed was efficient and rapid. Single-point mutation, deletion of large operons and consecutive deletion of multiple genes could be achieved easily. One plasmid and PCR cassette flanked by 50 bp homology regions were enough to induce efficient recombination, meaning only one step PCR amplification was needed. And as the marker cassettes could be used repeatedly, only the homology regions should be changed to perform the modifications of different genes, which may alleviate the workload of primer design.

Furthermore, the expression of the lambda Red proteins were driven by the tightly regulated promoter *P*_*BAD*_, of which the basal expression level was very low in the absence of its inducer. This will minimize the unwanted recombination and increase the efficiency of homologous recombination. On the other hand, the sucrose counter-selection procedure could also increase the selection efficiency. Therefore, the high recombination efficiency of this strategy could ease the screening step, lessen work intensity and shorten the experimental time.

Phenazine derivates have many important biological effects [31,32]. Although the pathway of phenazine synthesis in *P. aeruginosa *has been studied [33], the function mechanisms and regulation networks of phenazine derivates are still poorly characterized. Therefore, many knockout mutants need to be constructed, not only single gene mutant, but also the multiple-gene mutants. Based on plasmid pRKaraRed mediated method, we successfully obtained a series of scarless deletion mutants of different genes involving in the phenazine synthesis and regulation pathways, such as *lasI*, *qscR*, *gacA*, *rsmA *and *etc*. Using this scarless approach, mutants with modifications of multiple genes could be generated easily for further study of the cumulative effects in different combination styles. Strain PCA with the deletion in three genes was an example. It could be further used to study the regulation styles and the special functions of this compound without any disturbance of other phenazine derivates.

In a word, the plasmid pRKaraRed mediated method could perform efficient and accurate homologous recombination in *Pseudomonas *and in *E. coli*. There is only one potential shortcoming of this system, that this plasmid can not be removed easily after all the necessary modifications are accomplished. Therefore, further improvements may be done, such as using the conditional replicons (e.g. temperature-sensitive replicon) to perfect this system.

## Conclusion

This pRKaraRed-mediated technique could be used efficiently and rapidly to generate scarless and sequential gene modification mutants in *P. aeruginosa *with one-step PCR product flanked by short homology regions. Single-point mutation, large operon deletion mutants and sequential deletion mutants of multiple genes could be achieved easily. This method may give a new way to generate more genetically modified *P. aeruginosa *strains.

## Methods

### Strains, plasmids, enzymes and chemicals

All bacterial strains and plasmids used in this research were listed in Table 3. Luria-Bertani (LB) medium was used as a rich medium for both *E. coli *DH5α and *P. aeruginosa *PAO1. Phenazine compounds fermentation medium was PB (20 g/L Bacto Peptone, 1.4 g/L MgCl_2 _and 10 g/L K_2_SO_4_) [34]. The antibiotics carbenicillin (Carb, 500 μg/ml) and/or tetracycline (Tet, 50 μg/ml) were used if needed. 10% sucrose was used to identify the sucrose resistant or sensitive phenotype strain. Restriction enzymes, T4 DNA ligase, *LA-Taq *™ DNA polymerase, and *Pyrobest *™ DNA polymerase were purchased from TaKaRa BIOTECH Co. (Dalian, China). All other reagents and chemicals were of analytical grade.

**Table 3:** Bacterial strains and plasmids

Strains and Plasmids	Genotype or Description	Source
*E. coli *DH5α	*Sup E*44 ΔlacU169(Φ80 *lacZ*ΔM15) *hsd R*17 *recA*1 *endA*1*gyrA*96 *thi*-1 *rel A*1	Gibco-BRL

*P. aeruginosa *PAO1	Wild type prototroph	Stephen Lory's Lab

*P. aeruginosa *PCA	PAO1 Δ*phzH*Δ*phzS*Δ*phzM*	This work

Plasmid pDN18	RK2-derived cloning vector, Tet^R^	Stephen Lory's Lab, [18]

pBluescript II KS (+)	Universal cloning vector, Amp^R^	Stratagene

pEX18Ap	Gene replacement vector, *oriT*^+ ^*sacB*^+^, *Amp*^R^	Stephen Lory's Lab, [16]

pBAD18	Vector containing *araC *gene and *P*_*BAD *_promoter, Amp^R^	[35]

pRKaraRed	Broad-host-range, lambda Red proteins expression vector, Tet^R^	This work

### PCR and standard DNA procedure

PCR was performed with *LA-Taq *DNA polymerase or *Pyrobest *DNA polymerase according to the manufacyturer's protocol. DNA sequences of the oligonucleotides were listed in Additional file 1, Table S1. Oligonucleotides synthesis and DNA sequencing were performed by Invitrogen Ltd. (Shanghai, China). Plasmid DNAs were isolated using the QIA prep Mini-spin kit (Qiagen, Shanghai, China) and *P. aeruginosa *genomic DNA was obtained using QIA amp DNA mini kit (Qiagen, Shanghai, China). DNA fragment were purified from agarose gels utilizing the QIA quick gel extraction kit (Qiagen, Shanghai, China). Other general techniques for restriction enzyme manipulation, molecular cloning, and agarose gel electrophoresis were carried out with standard protocols.

### Construction of plasmid pRKaraRed

The cassette containing *araC *gene and *P*_*BAD *_promoter was amplified from plasmid pBAD18 with primers araF and araR (Additional file 1, Table S1) [35]. The amplified DNA fragments were digested with restriction enzymes *Kpn *I and *Xho *I, and then they were cloned into plasmid pBluescript II KS (+), generating plasmid pKS-ara. Similar method was used to amplify the three genes (*exo, bet *and *gam*) of lambda-Red recombination system from lambda phage genomes with primers RedF and RedR, and inserted it into the *Xho *I-*Bam *HI site of plasmid pKS-ara, yielding plasmid pKS-araRed. The *Kpn *I-*Bam *HI fragment containing *araC *gene, *P*_*BAD *_promoter and three *Red *genes was further sub-cloned into the *Kpn *I-*Bam*H I sites of RK2-derived cloning plasmid pDN18, generating the plasmid pRKaraRed able to express the lambda Red proteins (Fig. 1). DNA sequencing confirmed this construction.

### Electro-transformation of *P. aeruginosa*

Single *P. aeruginosa *colony was inoculated in 3 ml LB medium and grown at 37°C overnight. 1 ml overnight culture was added to 200 ml fresh LB medium and grown at 37°C, shaking to OD_600 _= 0.4~0.5. The bacteria were then rendered electro-competent by four times washings of ice-cold 10% glycerol and were re-suspended in 200 μl ice-cold 10% glycerol. To generate the electro-competent cells of PAO1/pRKaraRed, L-arabinose of certain concentration should be added into the medium and cultured for several hours before the 10% glycerol washing step.

Electroporation was carried out using 50 μl of bacterial suspension (about 1×10^9 ^cells) and no more than 10 μl of DNA (at least 200 ng/μl) in a 0.2 cm ice-cold electroportation cuvette, transformed on a Bio-Rad GenePulser II at 200Ω, 25 μF and 2.5 kV. Uninduced cells and induced cells without plasmid or without DNA fragments were set as negative controls for each transformation. The electroporated cells were diluted in 1 ml LB and incubated at 37°C for three hours. The transformants were then selected on the antibiotic-imbued plates.

### Scarless gene modification in *P. aeruginosa*

Scarless gene modification strategy was described in Fig. 2. First the *sacB*-*bla *cassettes were amplified from plasmid pEX18Ap with the primers F1 and R1 [16]. The numbers of primers corresponded to the steps of PCR amplification. The electro-transformation of the *sacB*-*bla *cassette into the PAO1/pRKaraRed competent cells was performed as described above. Transformants were screened on LB plates supplemented with 500 μg/ml carbenicillin and 50 μg/ml tetracyclin. The colonies with Carb^R^Tet^R ^phenotypes confirmed by PCR detection and DNA sequencing were regarded as positive clones. Next, the *sacB*-*bla *removal cassettes were amplified from the genomic DNA of the first-step strain with the primers F2 and R2. Then this fragment was electro-transformed into the competent cells of the first-step to perform the second recombination. Electro-transformed cells were spread on LB plates supplemented with 10% sucrose and 50 μg/ml tetracycline. The transformants were further selected parallel on the LB plates with 10% sucrose and 50 μg/ml tetracycline, and the LB plates with 500 μg/ml carbenicillin and 50 μg/ml tetracycline. The colonies with Suc^R^Carb^S ^phenotypes confirmed by PCR detection and DNA sequencing were regarded as positive recombinants.

Twelve genes, two large operons and one nucleotide site were selected as target and their primers for PCR amplification were listed in Additional file 1, Table S1.

### System efficiency analysis

The influences of L-arabinose concentration, induction time and the length of homology region on the efficiency of homologous recombination were analyzed. *phzS *gene was selected as target. First, the PAO1/pRKaraRed cultures were induced with L-arabinose of different concentrations (ranging from 0.05% to 1.0%) for three hours. Then the PAO1/pRKaraRed cultures were induced with L-arabinose of suitable concentration for different time (from 1 h to 12 h). Finally, the PCR products with homology regions of different lengths (50 bp, 60 bp, 100 bp) were used to perform homologous recombination. Control experiments and screen procedures were set same as described above. The efficiencies of recombination were calculated by dividing the number of positive colonies with the number of growing colonies.

### Construction of three-gene deleted strain PCA and HPLC analysis of phenazine derivatives

Sequential gene modifications of multiple target genes were achieved by several rounds of recombination steps. The recombination efficiency was also detected using phenotype screen, PCR detection and DNA sequencing. The strain with three-gene deletions (PAO1, *ΔphzHΔphzMΔphzS*) was named as PCA.

HPLC analysis of phenazine derivatives were performed as previously described [33]. PAO1 and PCA strains were cultured in PB medium at 28°C for 72 h and then centrifugation was performed to remove the cells. The recovered medium was acidified to pH 4.0 with HCl and filtered through 0.22 μm membrane. The filtrates were extracted with chloroform. The organic phase was dried with nitrogen and dissolved in acetonitrile. 10 μl samples were loaded onto a Unimicro Kromasil C18 column (5 μm; 4.6 by 250 mm, ScienHome Co., USA) for reverse-phase HPLC analysis in a Waters HPLC Integrity system consisting of a Waters 510 separation module and a 490E programmable multi-wavelength detector. The column was washed at a flow rate 500 μl/min with 8% acetonitrile in 25 mM ammonium acetate for 2 min and a linear gradient acetonitrile from 8% to 80% in 25 mM ammonium acetate for 25 min. The HPLC was monitored simultaneously at 257 nm. The peak fractions were collected separately and identified by mass spectrometry with HP1100 HPLC-MSD (API-ES/APCI) (Hewlett-Packard Co., USA).

## Authors' contributions

RL conceived of the study, carried out all the molecular genetic studies and HPLC analysis, participated in the sequence alignment and drafted the manuscript. JL conceived of the study, participated in its design and coordination.

All authors have read and approved the final manuscript.

## Supplementary Material

Additional file 1 **Table S1 - Oligonucleotides used for PCR amplifications**. Click here for file
