# Supplementary material for: Scarless and sequential gene modification in Pseudomonas using PCR product flanked by short homology regions
Source: BMC Microbiol. 2010 Aug 3;10:209. doi: 10.1186/1471-2180-10-209 (PMC2924854; doi:10.1186/1471-2180-10-209)
Supplement: Additional file 1 — Table S1 - Oligonucleotides used for PCR amplifications. [file 1471-2180-10-209-S1.DOC]

## Table S1. Oligonucleotides used for PCR amplifications

| Oligos* | Sequences # | Usage |
| --- | --- | --- |
| araF | 5’ggggggggtaccttatgacaacttgacggctacatca3’ | *araC* gene and *P_BAD_* promoter |
| araR | 5’ggggggctcgagccaccaacagtagagagttgcgataaaaagcgt3’ |
| RedF | 5’ggggggctcgagatggatattaatactgaaactgagatcaag 3’ | *Red* genes (*gam*, *bet*, *exo*) |
| RedR | 5’ggggggggatcctcatcgccattgctccccaaa 3’ |
| PhzH F1 | 5’atgagtttcccgataaacatcaattataggagtttccctaggatcgatcctttttaaccc3’ | *sacB*-*bla* cassette targeting *phzH* |
| PhzH R1 | 5’gccggacagcagcccgaacccgccacgccccgcgtcacgctgagtaaacttggtctgac3’ |
| PhzH F2 | 5’atgagtttcccgataaacatcaattataggagtttccctagcgtgacgcggggcgtggcg3’ | *sacB*-*bla* removal fragment of *phzH* |
| PhzH R2 | 5’ccggagtctgggccaggatg3’ |
| PhzH DF | 5’atgagtttcccgataaacat3’ | Detection primers |
| PhzH DR | 5’gccggacagcagcccgaacc3’ |
| PhzS F1 | 5’cgacgaaggaggctggaagcaccgctacctgcaaccgtgaggatcgatcctttttaaccc3’ | *sacB*-*bla* cassette targeting *phzS* |
| PhzS R1 | 5’gaggcgggagagggcaggcgcggtggcctgcccggtgttgagtaaacttggtctgac3’ |
| PhzS F1 | 5’cgacgaaggaggctggaagcaccgctacctgcaaccgtgcaacaccgggcaggccaccg3’ | *sacB*-*bla* removal fragment of *phzS* |
| PhzS R2 | 5’aggtcaacgcggtacagatc3’ |
| PhzS DF | 5’cgacgaaggaggctggaagca3’ | Detection primers |
| PhzS DR | 5’gaggcgggagagggcaggc3’ |
| PhzM F1 | 5’ttctcgggttacggaaatgtgtaacgagagagaataaaagatgaacatcaaaaagtttgc3’ | *sacB*-*bla* cassette targeting *phzM* |
| PhzM R1 | 5’gccggcgaaaccgccggggccaaccgttgaaagttccgatagttaccaatgcttaatcag3’ |
| PhzM F2 | 5’ttctcgggttacggaaatgtgtaacgagagagaataaaagatcggaactttcaacggttg3’ | *sacB*-*bla* removal fragment of *phzM* |
| PhzM R2 | 5’tgggaaatcgacctgttcgg3’ |
| PhzM DF | 5’ttctcgggttacggaaatgtg3’ | Detection primers |
| PhzM DR | 5’gccggcgaaaccgccggggc3’ |
| lasI F1 | 5’attatgaaatttgcataaattcttcagcttcctatttggaggaagtgaagatgaacatcaaaaagtttgc3’ | *sacB*-*bla* cassette targeting *lasI* |
| lasI R1 | 5’gcgctccctctccgacagagaacgcgccggcgcgagccgacaggtccccgagttaccaatgcttaatcag3’ |
| lasI F2 | 5’attatgaaatttgcataaattcttcagcttcctatttggaggaagtgaagcggggacctgtcggctc3’ | *sacB*-*bla* removal fragment of *lasI* |
| lasI R2 | 5’agcgatctcacgccggtcgcctatct3’ |
| lasI DF | 5’cagcttcctatttggaggaagtgaa3’ | Detection primers |
| lasI DR | 5’ gagccgacaggtccccg3’ |
| rhlR F1 | 5’ccccgggccaattctgctgtgatgcattttatcgatcagggcttactgcaatgaacatcaaaaagtttgc3’ | *sacB*-*bla* cassette targeting *rhlR* |
| rhlR R1 | 5’aggcaacctgccagatctggtagggcgcgccgaccggcgcgccctgcgctagttaccaatgcttaatcag3’ |
| rhlR F2 | 5’ccccgggccaattctgctgtgatgcattttatcgatcagggcttactgcaagcgcagggcgcgccgg3’ | *sacB*-*bla* removal fragment of *rhlR* |
| rhlR R2 | 5’cagcagcgcaaggcaggcca3’ |
| rhlR DF | 5’atcgatcagggcttactgca3’ | Detection primers |
| rhlR DR | 5’aggcaacctgccagatct3’ |
| gacA F1 | 5’cctgggggcccgtgtgggcggttgtctgctaatgcgcgacgaggtgcagcatgaacatcaaaaagtttgc3’ | *sacB*-*bla* cassette targeting *gacA* |
| gacA R1 | 5’cgattgctacaggtagcgaggaaggcgctcgcgtcgaaaacggcgctcatagttaccaatgcttaatcag3’ |
| gacA F2 | 5’cctgggggcccgtgtgggcggttgtctgctaatgcgcgacgaggtgcagcatgagcgccgttttcgacgc3’ | *sacB*-*bla* removal fragment of *gacA* |
| gacA R2 | 5’atcgccgaaaccagcaccgg3’ |
| gacA DF | 5’aatgcgcgacgaggtgcagc3’ | Detection primers |
| gacA DR | 5’gcgtcgaaaacggcgctcat3’ |
| qscR F1 | 5’gctgaagaaccgaatcctgccgggatcgattgttgactggtgaagctggcatgaacatcaaaaagtttgc3’ | *sacB*-*bla* cassette targeting *qscR* |
| qscR R1 | 5’gaatggccggacgcttgcgcggcggcctcgctaggcgacgaggcgtcgatagttaccaatgcttaatcag3’ |
| qscR F2 | 5’gctgaagaaccgaatcctgccgggatcgattgttgactggtgaagctggcatcgacgcctcgtcgcctag3’ | *sacB*-*bla* removal fragment of *qscR* |
| qscR R2 | 5’tcaggtgggaataccgtcac3’ |
| qscR DF | 5’cgattgttgactggtgaagctggc3’ | Detection primers |
| qscR DR | 5’ctaggcgacgaggcgtcgat3’ |
| rsmA F1 | 5’tttcttttttgcagactgttgtcctgaaatattcgcgtgaggagaaaggaatgaacatcaaaaagtttgc3’ | *sacB*-*bla* cassette targeting *rsmA* |
| rsmA R1 | 5’cgcatgatacccatctttaccccgtttgcaaagggaaaattagataaaaaagttaccaatgcttaatcag3’ |
| rsmA F2 | 5’tttcttttttgcagactgttgtcctgaaatattcgcgtgaggagaaaggatttttatctaattttccctttgcaaacggg3’ | *sacB*-*bla* removal fragment of *rsmA* |
| rsmA R2 | 5’agcacggtgatcctgcagac3’ |
| rsmA DF | 5’attcgcgtgaggagaaagga3’ | Detection primers |
| rsmA DR | 5’cgtttgcaaagggaaaattagataaaaa3’ |
| ptsP F1 | 5’ggccccgcgccttctggcgcgggactgaacacggagacaaggccccgagcatgaacatcaaaaagtttgc3’ | *sacB*-*bla* cassette targeting *ptsP* |
| ptsP R1 | 5’tgcggggaaagcgtaagcgccgcacgacgaagcgtccggcgcgcgggaaaagttaccaatgcttaatcag3’ |
| ptsP F2 | 5’ggccccgcgccttctggcgcgggactgaacacggagacaaggccccgagctttcccgcgcgccggacg3’ | *sacB*-*bla* removal fragment of *ptsP* |
| ptsP R2 | 5’cgctggcgcaggcgttcgg3’ |
| ptsP DF | 5’ggagacaaggccccgagc3’ | Detection primers |
| ptsP DR | 5’ gtccggcgcgcgggaaa3’ |
| lasR F1 | 5’cgaatccatatttggctgattggttaatagtttaagaagaacgtagcgctatgaacatcaaaaagtttgc3’ | *sacB*-*bla* cassette targeting *lasR* |
| lasR R1 | 5’tatagaagggcaaattaccgatcgccagctcgccgacctgagaggcaagaagttaccaatgcttaatcag3’ |
| lasR F2 | 5’cgaatccatatttggctgattggttaatagtttaagaagaacgtagcgcttcttgcctctcaggtcggcg3’ | *sacB*-*bla* removal fragment of *lasR* |
| lasR R2 | 5’acaggtccccgtcatgaaac3’ |
| lasR DF | 5’agtttaagaagaacgtagcgct3’ | Detection primers |
| lasR DR | 5’ gccgacctgagaggcaaga3’ |
| rpoD F1 | 5’aatcctctgcttattttttgcccgccaagaccttcagtggatagggtgttatgaacatcaaaaagtttgc3’ | *sacB*-*bla* cassette targeting *rpoD* |
| rpoD R1 | 5’ggcaagcggcaaagaaaaacccccggtttttcaggccgggggttcgttcgagttaccaatgcttaatcag3’ |
| rpoD F2 | 5’aatcctctgcttattttttgcccgccaagaccttcagtggatagggtgttcgaacgaacccccggcc3’ | *sacB*-*bla* removal fragment of *rpoD* |
| rpoD R2 | 5’ccggcgactcacctcgcggc3’ |
| rpoD DF | 5’accttcagtggatagggtgtt3’ | Detection primers |
| rpoD DR | 5’ggtttttcaggccgggg3’ |
| rpoS F1 | 5’gggaaaggaatcgcccgggcttgagtcgaactcatgcaagggataacgacatgaacatcaaaaagtttgc3’ | *sacB*-*bla* cassette targeting *rpoS* |
| rpoS R1 | 5’cacaaaaaacccggcgaacgccgggtcttcagtgggtctaaggttttccgagttaccaatgcttaatcag3’ |
| rpoS F2 | 5’gggaaaggaatcgcccgggcttgagtcgaactcatgcaagggataacgaccggaaaaccttagacccact3’ | *sacB*-*bla* removal fragment of *rpoS* |
| rpoS R2 | 5’cccgcgaacacctcaag3’ |
| rpoS DF | 5’cgaactcatgcaagggataacgac3’ | Detection primers |
| rpoS DR | 5’ttcagtgggtctaaggttttccg3’ |
| phzS761 F1 | 5’catggtgccgagcgccgccgtcggccagctcgacaacgaggccgactggaatgaacatcaaaaagtttgc3’ | *sacB*-*bla* cassette targeting *phzS761* site |
| phzS761 R1 | 5’ggtcccagtcggcgaagaacggcagcacgtcctccaggcgcccgtcgcggagttaccaatgcttaatcag3’ |
| phzS761 F2 | 5’catggtgccgagcgccgccgtcggccagctcgacaacgaggccgactggaccgcgacgggcgcctgga3’ | *sacB*-*bla* removal fragment of *phzS761* |
| phzS761 R2 | 5’atcacctcggtggcgatcct3’ |
| phzS761 DF | 5’cgacaacgaggccgactgga3’ | Detection primers |
| phzS761 DR | 5’tccaggcgcccgtcgcgg3’ |
| PhzA1G1 F1 | 5’ttctccggcatacctggagagccctctcggaggcggcgcggatcgatcctttttaaccc3’ | *sacB*-*bla* cassette targeting *phzA1G1* operon |
| PhzA1G1 R1 | 5’cgccggtggcgtcattcgccctacgaaccggctccgaggctgagtaaacttggtctgac3’ |
| PhzA1G1 F2 | 5’ttctccggcatacctggagagccctctcggaggcggcgcgcctcggagccggttcgtaggg3’ | *sacB*-*bla* removal fragment of *phzA1G1* operon |
| PhzA1G1 R2 | 5’tccaggcgcccgtcgcgg3’ |
| PhzA1G1 DF | 5’cctctcggaggcggcgc3’ | Detection primers |
| PhzA1G1 DF | 5’ctacgaaccggctccgaggc3’ |
| PhzA2G2 F1 | 5’atggatgccagtcgattcgaactggcggagattcgcaccggatcgatcctttttaaccc3’ | *sacB*-*bla* cassette targeting *phzA2G2* operon |
| PhzA2G2 R1 | 5’gtccaaccccgaacatcgtctgggccggactccgagtccgtgagtaaacttggtctgac3’ |
| PhzA2G2 F2 | 5’atggatgccagtcgattcgaactggcggagattcgcacccggactcggagtccggcccaga3’ | *sacB*-*bla* removal fragment of *phzA2G2* operon |
| PhzA2G2 R2 | 5’ccggctgttcggcatcgaaccgcc3’ |
| PhzA2G2 DF | 5’aactggcggagattcgcacc3’ | Detection primers |
| PhzA2G2 DF | 5’gccggactccgagtccg3’ |

* The oligonucleotides were named according to the gene name and their use. Primers F1 and R1 were used for the first-step amplification of the *sacB*-*bla* cassette with 50bp homology, and the primers F2 and R2 were for the second-step amplification of the *sacB*-*bla* removal fragment. The primers DF and DR were used for the detection of target gene modification.

# The underlined sequences indicated the restriction sites for DNA cloning and the shaded sequences denoted the 50bp homology region flanking the targets.
